# The prevalence and associated risk factors of post-transplant *Toxoplasma gondii* infection among kidney transplant recipients and patients with uremia in Central-southern China

**DOI:** 10.1371/journal.pntd.0013063

**Published:** 2025-05-30

**Authors:** Xingxing Zheng, Junhui Li, Zhuolin Li, Xianshu Liu, Yufei Zhang, Jiang Zhu, Yu Zhang, Jie Jiang, Bo Li, Meng Xia, Yingzi Ming, Xiang Wu

**Affiliations:** 1 Department of Parasitology, Xiangya School of Basic Medicine, Central South University, Changsha, Hunan, China; 2 Organ Tranplant Center, 3rd Xiangya Hospital of Central South University, Changsha, Hunan, China; 3 Hunan Province Clinical Research Center for Infectious Diseases, Changsha, Hunan, China; 4 Key Laboratory of Translational Research on Transplantation Medicine of National Health Committee, 3rd Xiangya Hospital of Central South University, Changsha, Hunan, China; 5 Hunan Provincial Key lab of Immunology and Transmission Control on Schistosomiasis (The Third People’s Hospital of Hunan Province), Yueyang, China; U.S. Food and Drug Administration and Center for Biologics Evaluation and Research, UNITED STATES OF AMERICA

## Abstract

**Background:**

*Toxoplasma gondii (T. gondii)* is an opportunistic intracellular parasite *and* is a big threaten for patients with uremia and solid organ transplantation recipients, especially for kidney transplant recipients. However, *Toxoplasma* seroprevalence in these patient populations remain unclear, and the risk factors of post transplantation *T. gondii* infection were not well-defined in China and globally.

**Methods:**

Post-transplant or uremia patient’s serum collected from transplant center of 3^rd^ Xiangya hospital of Central South University were detected by IHA to detect anti-*Toxoplasma* IgG. A retrospective cross-sectional study was performed with these patient’s medical records and a questionnaire was also conducted.

**Results:**

The results indicated that approximately 10.59% of the kidney recipients in central-southern China were seropositive for *T. gondii* post-transplant. While the prevalence in patients with uremia was about 24.12%. We identified that keeping cats and a lower percentage of CD3+T cells and CD8+T cells were associated with higher prevalence of *T. gondii* IgG+ in uremia patients when compared with kidney transplant recipients.

**Conclusions:**

This study suggested that kidney recipients and those uremia patients in the waiting list are susceptible to *T. gondii* infection. Immune status and keeping cats are associated with the seroprevalence in those patients. However, *T. gondii* infection is a neglected problem, screening and monitoring deserves more attention in the clinic.

## Author summary

*Oxoplasma gondii* is an opportunistic intracellular parasite *and* is Serious threaten for immunodeficient patients including those with uremia and solid organ transplantation recipients. However, *Toxoplasma* seroprevalence in these patients remains unclear, and the risk factors of post transplantation *T. gondii* infection were not well defined in China and globally. Therefore, *Toxoplasma* infection is still a neglected problem, and clinical screening and monitoring should be pay more attention. This study suggested that kidney recipients and those uremia patients in the waiting list are susceptible to *T. gondii* infection. Immune status and keeping cats are associated with the seroprevalence in those patients.

## Introduction

*Toxoplasma gondii* is a protozoan parasite capable of infecting almost all warm-blooded animals and humans. It is estimated that about 30% of the population in the world are affected by *T. gondii* [1]. *T. gondii* infection is usually caused by ingestion of raw or undercooked meat with viable tissue cysts, or by eating oocysts contaminated food from infected cat feces. Although it is often asymptomatic in immunocompetent individuals, *T. gondii* infection is an opportunistic infection and can cause severe disease in immunocompromised individuals, including patients with immunosuppressant use, those received organ or bone morrow transplantation, and HIV infection [2–6]. For immunocompromised hosts, infection with *T. gondii* can lead to encephalitis, pneumonitis, and other serious complications, which can lead to high morbidity and mortality [2–6]. However, the risk factors have been difficult to characterize and the diagnosis far from satisfaction.

Solid organ transplant recipients are facing high risk of *T. gondii* infection for long-term use of immunosuppressants. Post-transplant toxoplasmosis can be transmitted by donor infections or result from reactivation of latent infections because of immunosuppression [7]. With marked shortage of organ donors, more and more suboptimal organs are accepted in the clinical practice, including organs from *T. gondii* antibody-positive donors. However, the risk of toxoplasmosis is higher in transplantation of an organ from seropositive donor into seronegative recipient than that of transplanting a non-infected organ into a seropositive patient [6,8–10]. Moreover, fatal toxoplasmosis has been reported in heart, liver, kidney transplant recipients [8,11]. A retrospective study showed that the mortality rate of solid organ transplant recipients with toxoplasmosis was 13.6% [9]. Therefore, there are some guidelines recommending *T. gondii* screening in both donors and recipients, although they leave the decision of transplantation to individual center [11,12].

Among solid organ transplantation, kidney transplantation recipients are at high risk because of increased severity of immunosuppression. Post-kidney-transplant toxoplasmosis can result from reactivation of latent infection for immunosuppression, as uremia patients are susceptible to *T. gondii* infection [13–17]. There were evidences suggested that toxoplasmosis in kidney transplant recipients could be transmitted by organs from infected donors [18,19]. Therefore, it is also a potential threat. The seroprevalence of *T. gondii* varies dramatically by location, but the seroprevalence of *T. gondii* and the incidence of post-transplant toxoplasmosis before and after kidney transplant remains unclear. In addition, studies have identified various risk factors for toxoplasmosis, including age, cat-keeping, raw meat-eating habits, and raw meat handling [6,20]. But the risk factors of *Toxoplasma* infection before and after kidney transplant in China is still not well-known.

In this study, we retrospectively studied the seroprevalence of *T. gondii* infection in patients received kidney transplantation and uremia patients in the waiting list of kidney transplantation. We also explored the associated risk factors and the impact of post-transplant *T. gondii* infection on transplant recipients.

## Materials and methods

### Ethics statement

This study was approved by the Institutional Review Board (IRB) of The Third Xiangya Hospital of Central South University (Fast 24914). All participants provided were informed about the study’s purpose, procedures, potential risks, and benefits. Participants were assured of confidentiality, and data were anonymized prior to analysis. For minors under 18 years old, consent was obtained from their parents or legal guardians.

### Study design

This single-center retrospective cross-sectional study was conducted at the 3^rd^ Xiangya Hospital of Central South University, located in Changsha City of Hunan province, a place in central-southern China. All patients (≥ 18 years old) received kidney transplant and those with uremia who visited the 3rd Xiangya Hospital of Central South University, from May 2023 to May 2024, were recruited. The patients who took immune status assessment and the screening test for the IgG antibody specific for *T. gondii* simultaneously were included in the study. The exclusion criteria included: (1) history of secondary or multiple renal transplantation;(2) history of other solid organ or tissue transplantation; (3) history of infection within a week; (4) history of cancer. Healthy volunteers were recruited from those who received health examination in the same hospital. According to the inclusion and exclusion criteria, 576 patients received kidney transplantation, 257 patients with uremia, as well as 518 healthy volunteers were included. The clinical information of the patients with kidney transplantation or uremia in the hospital were collected. The study was reviewed and approved by the Institutional Review Board of Third Xiangya Hospital, Central South University.

### Immune status assessment

The immune status assessment was performed with BD Multi-test 6-color TBNK reagent with BD Trucount tubes according to manufacturer’s instructions (BD Biosciences, USA), determining the percentages and absolute counts of peripheral immune cell subsets, including CD4+ T cells, CD8+ T cells, NK cells as well as CD19+ B cells. BD TruCOUNT Tubes (BD Biosciences) was filled with 50 µl anticoagulant whole blood, then a monoclonal antibody mixture (20 µl) was added and incubated for 15 min in the dark. After washing and resuspending, samples were analyzed by flow cytometer (BD FACSCanto II) and the data were analyzed using the BD FACSCanto clinical software (BD Biosciences, San Jose, CA).

### Anti-*T. gondii* IgG antibody detection

Anti*-T. gondii* IgG antibody (Tg-IgG) was detected via indirect hemagglutination test (IHA) kit according to manufacturer’s instructions (Lanzhou Shouyan Biotechnology, Lanzhou, China). Serum was diluted in 1:64 and 1:128 with dilutions on 96-well V-bottom microwell plates and incubated at 37°C for 1 hours after adding antigen diagnostic solution. The positive serum had reactions obtained at 1:64 or greater dilutions.

### Statistical analysis

Quantitative variables were represented as mean ± standard deviation (SD). Differences between groups were compared using Fisher’s exact test or Pearson’s chi-squared (*χ*^2^) test, as appropriate, for categorical data, while continuous data were analyzed using the t test. Statistical analysis was conducted with GraphPad Prism version 10.1.2 for Windows (GraphPad Software, San Diego, CA USA). The P value of <0.05 was considered as statistically significant.

## Results

### Demographic characteristics of the study participants

According to our including and excluding criteria, this study included 576 kidney transplant recipients, 257 patients with uremia and 518 healthy volunteers (Table 1). Among the kidney recipients, 363 (63.02%) were male, and the average age was 44.91± 10.66 years (Table 1). In the group of patients with uremia, 154 (59.92%) were male, and the average age was 43.30±10.92 years (Table 1). Meanwhile in healthy control group, 305(58.88%) were male and the average age was 43.36±15.59(Table 1). No significant differences were observed in age or sex among the three groups (Table 1).

**Table 1:** Demographic characteristics of the participants.

Parameters	Healthy Ctr	Renal Tx	Uremia	P value
**Age, years ± SD**	43.36 ± 15.59	44.91 ± 10.66	44.30 ± 10.92	0.4082
**Male, n (%)**	305 (58.88%)	363 (63.02%)	154 (59.92%)	0.3517
**Female, n (%)**	213 (41.12%)	213 (36.98%)	103 (40.08%)	

Renal Tx: Renal transplant recipients

### The serostatus of *T. gondii* infection

The serum of all the participants were used for *Tg*-IgG test. The serostatuses of the recipients, patients with uremia and healthy volunteers were presented in Table 2. In the group of kidney recipients, 61 out of 576 (10.59%) were seropositive (Table 2). Among the healthy volunteers, only 9 individuals were positive, a very low percentage according to previous studies [21]. However, the percentage of seropositive patients with uremia was 24.12%, significantly higher when compared with any other group (Table 2).

**Table 2:** The seroprevalence of anti-*Toxoplasma* antibodies IgG.

	*Tg*-IgG(−)	*Tg*-IgG(+)	P value
**Healthy Ctr**	509 (98.26%)	9 (1.74%)	
**Renal Tx**	515 (89.41%)	61 (10.59%)	<0.001
**Uremia**	195 (75.88%)	62 (24.12%)	

*Tg*-IgG: anti-*Toxoplasma* antibodies IgG

### The differences in *T. gondii* seropositivity between uremia patients and kidney recipients

It was interesting that patients received kidney transplantation had a lower percentage of seropositive status compared to those uremia patients, because previous studies demonstrated immunosuppression was an independent risk factor of *T. gondii* infection. To explore the associated factors, we performed a questionnaire survey. 156 kidney recipients and 71 patients with uremia answered the questionnaire on their living condition and potential exposure. The questionnaire survey results were shown in Table 3. We noticed that there was no significant difference in residency, education, or occupational status between patients received renal transplantation and those with uremia. However, the percentage of raising pets was higher in patients with uremia than those with renal transplantation (Table 3). Thus, the result suggested that raising pets was associated with higher percentage of seropositivity of *T. gondii* infection in patients with uremia, which was also reported as a risk factor of *T. gondii* infection. Although raw-meat eating habits and meat-handling work can increase the risk of *T. gondii* infection, no differences were found between the two groups in terms of raw meat eating habits and meat handling jobs (Table 3).

**Table 3:** Residence, education, occupational status, raising pets, raw-meat eating habit and raw-meat handling work between kidney transplant recipients and uremia patients.

	Renal Tx	Uremia	P value
**Residency**			
City	105	51	
Suburb	10	3	0.7525
Rural area	41	17	
**Education**			
Below undergraduate	116	47	
Undergraduate	36	22	0.4170
Graduate	4	2	
**Occupational status**			
Employed	69	22	
Retired	33	13	0.0659
Unemployed	54	36	
**Raising pets**	40	30	0.0120
**Raw-meat eating habits**	12	4	0.5743
**Raw-meat-handling work**	26	14	0.5775

It is well-recognized that immune status plays a crucial role in *T. gondii* infection. So we also analyzed the immune status of patients to investigate whether the difference in the percentage or absolute number of T cells, B cells and NK cells were associated with high risk of *T. gondii* infection in uremia. As was shown in Table 4, the percentages of CD3+T cells and CD8+T cells in uremia patients were lower than those in kidney transplant patients, but the percentage of CD4+T cells was higher in patients with kidney transplantation (Table 4). However, there was no significant difference in the absolute number of CD3+T cells, CD4+T cells and CD8+T cells between the two groups (Table 4). Additionally, no difference was observed in the percentage or absolute number of NK cells (Table 4). This may be due to that these tested patients maintained relative stable immunity, or those patients with acute *T. gondii* infection was not included for death and without detection, or part of immunocompromised transplant patients or uremic patients unable to produce sufficient antibodies for impaired humoral immunity [14,22–24]. Interestingly, patients with uremia had higher number of CD19+B cells, even though the percentage was similar (Table 4).

**Table 4:** The immune status between patients with renal transplantation and those with uremia.

	Renal Tx	Uremia	P value
CD3^ +^ T cells/TBNK, mean ± SD (%)	74.03 ± 11.95	72.16 ± 8.915	0.0066
CD3^ +^ T cells, *n* ± SD (cells/µl)	1036 ± 580.3	1062 ± 534.7	0.7477
CD8^ +^ T cells/TBNK, mean ± SD (%)	32.57 ± 11.08	26.32 ± 8.815	<0.001
CD8^ +^ T cells, *n* ± SD (cells/µl)	451.2 ± 227.6	404.6 ± 251.2	0.2309
CD4^ +^ T cells/TBNK, mean ± SD (%)	37.11 ± 11.64	41.90 ± 7.163	0.0017
CD4^ +^ T cells, *n* ± SD (cells/µl)	535.7 ± 336.1	615.5 ± 297.4	0.0885
NK cells/TBNK, mean ± SD (%)	13.29 ± 8.926	14.91 ± 8.108	0.1948
NK cells, *n* ± SD (cells/µl)	193.0 ± 197.5	199.6 ± 134.1	0.7987
B cells/TBNK, mean ± SD (%)	10.48 ± 10.29	11.53 ± 6.053	0.4259
B cells, *n* ± SD (cells/µl)	128.0 ± 103.7	144.7 ± 80.79	0.2320
CD4/CD8 ratio, mean ± SD	1.370 ± 0.8637	1.625 ± 0.5553	0.0298

### Associated factors of post-transplant *T. gondii* seropositivity

*T. gondii* infection was a serious condition for patients with organ transplantation for immunosuppression, thus identifying risk factors and associated factors is of great significance. Although acute toxoplasmosis in post kidney transplantation patients is rare, *T. gondii* seropositivity is common and deserves more attention. To explore the associated factors of post-transplant *T. gondii* seropositivity, we further analyzed the differences between those with *Tg*-IgG+ and those with *Tg*-IgG-. As was presented in Table 5, no difference was observed in age (Table 5). However, we found that the percentage of male participants declined in patients with *Tg*-IgG- (Table 5). Also, we performed analysis on biochemical testing data. The liver function data showed that both the level of glutamic pyruvic transaminase (ALT) and glutamic oxaloacetic transaminase (AST) was similar between the two groups, meanwhile the renal function results indicated that *Tg*-IgG+ group had a similar level of blood urea nitrogen (BUN), creatinine (Cr) and uric acid UA (Table 5). Besides, we evaluated their immune status to detect the impact of immune cells on *Tg*-IgG+. Immune analysis indicated that *Tg*- IgG+ group had a similar percentage of CD3+T cells, CD4+T cells, CD8+T cells, NK cells and B cells with *Tg*-IgG- group (Table 6). Additionally, there was no significant differences in absolute number of CD3+T cells, CD4+T cells, CD8+T cells, NK cells and B cells between the two groups (Table 6). Meanwhile, the ratio of CD4/CD8 in the group of *Tg*-IgG- was comparable with that in the group of *Tg*-IgG+ (Table 6).

**Table 5:** Clinical characteristics of patients received renal transplant.

Parameters	*Tg*-IgG(+)	*Tg*-IgG(-)	P value
Age, years ± SD	42.80 ± 10.97	45.25 ± 10.52	0.0879
Male, n (%)	48 (78.69%)	315 (61.17%)	0.0075
Female, n (%)	13 (21.31%)	200 (38.83%)	
BUN(mmol/L), mean ± SD	7.313 ± 3.556	8.390 ± 4.804	0.0909
Cr(umol/L), mean ± SD	106.3 ± 35.15	121.0 ± 72.48	0.1752
UA(umol/L),mean ± SD	372.4 ± 85.03	352.2 ± 91.75	0.1575
ALT(U/L), mean ± SD	22.91 ± 24.37	18.82 ± 17.83	0.1773
AST(U/L),mean ± SD	20.55 ± 9.569	21.64 ± 24.72	0.7664
WBC(109/L), mean ± SD	7.286 ± 2.175	7.235 ± 2.558	0.8961
Hb(g/L), mean ± SD	131.05 ± 28.04	124.5 ± 24.13	0.0717
Plt(109/L), mean ± SD	216.0 ± 64.75	205.0 ± 62.80	0.2649

BUN: blood urea nitrogen; Cr: creatinine; UA: uric acid; ALT: alanine aminotransferase; AST: Aspartate Aminotransferase; WBC: white blood cells; Hb: Hemoglobin; Plt: platelet.

**Table 6:** The immune status between kidney recipients with *Tg*-IgG+ and those with *Tg*-IgG-.

	*Tg*-IgG(+)	*Tg*-IgG(-)	P value
CD3^ +^ T cells/TBNK, mean ± SD (%)	79.40 ± 12.13	73.14 ± 11.54	0.4533
CD3^ +^ T cells, *n* ± SD (cells/µl)	1238 ± 635.9	1050 + 582.3	0.0565
CD8^ +^ T cells/TBNK, mean ± SD (%)	31.44 ± 9.961	32.34 ± 10.43	0.6093
CD8^ +^ T cells, *n* ± SD (cells/µl)	554.4 ± 402.3	466.4 ± 266.4	0.0664
CD4^ +^ T cells/TBNK, mean ± SD (%)	38.13 ± 9.721	37.01 ± 11.30	0.5470
CD4^ +^ T cells, *n* ± SD (cells/µl)	634.0 ± 328.8	554.0 ± 330.5	0.1498
NK cells/TBNK, mean ± SD (%)	12.63 ± 8.828	14.38 ± 9.292	0.2603
NK cells, *n* ± SD (cells/µl)	210.5 ± 169.5	208.5 ± 192.4	0.9504
B cells/TBNK, mean ± SD (%)	9.789 ± 7.568	10.37 ± 8.520	0.6789
B cells, *n* ± SD (cells/µl)	158.9 ± 120.2	137.1 ± 97.31	0.1957
CD4/CD8 ratio, mean ± SD	1.458 ± 0.8480	1.322 ± 0.7173	0.2673

These results suggest that the maintenance of immunity and normal function in kidney transplant patients is vital for successful transplantation in patients with *T. gondii* positive. Because *T. gondii* is an opportunistic parasite, it can cause fatal consequences in immunocompromised hosts, especially where cellular immunity is important.

## Discussion

Transplant recipients belong to typical immunocompromised individuals because of long-term use of immunosuppressants, which makes them at high risk of acute infection or reactivation of latent infection of *T. gondii* [25,26]. The higher percentage of seropositivity of Tg-IgG in patients received kidney transplant or in the waiting list indicated increased threaten of *T. gondii* infection for them. Furthermore, toxoplasmosis will lead to morbidity and mortality, especially for those with severe immunosuppression [26]. Toxoplasmosis will also increase the risk of graft failure and even death.

In this retrospective study from a transplant center in central-southern China, we found that approximately 10.59% of the kidney recipients were seropositive for *T. gondii* post-transplant, and the prevalence in patients with uremia was about 24.12%. We identified that raising pets and lower percentage of CD3+T cells and CD8+T cells was associated with higher prevalence of *Tg*-IgG+ in uremia patients when compared with that in kidney transplant recipients. Nonetheless, no significant difference was observed in blood routine, biochemical test as well as immune status between kidney recipients with *Tg*-IgG+ and *Tg*-IgG-. This study supplied more evidence in the prevalence of post-transplant *T. gondii* seropositivity and explored the associated risk factors, which will contribute to the understanding of post-transplant *T. gondii* infection and arise attention in the clinic.

This prevalence in kidney recipients was largely in line with that of other populations in previous reports in China [27–29]. Among those immune suppressed patients, such as hematopoietic stem cell transplant recipients, the pre-transplant seroprevalence was 13.8%, and the incidence of toxoplasmosis was 3.2% [30]. For solid organ transplantation patients, most studies focused on the prevalence of toxoplasmosis but not the seropositivity. The prevalence of toxoplasmosis was 0.14%, while the prevalence of seropositive for *T. gondii* was much higher in solid organ transplantation (SOT) recipients [6,31]. This phenomenon suggested *T. gondii* seropositivity is possibly a neglected problem and more study is warranted. Immunosuppression is a key factor for seropositivity post transplantation, thus the higher seroprevalence in the kidney recipients suggested that rational and precise use of immunosuppressants is of great significance.

In this study we explored the associated risk factor of post-transplant *T. gondii* seropositivity and our results seemed consistent with previous studies [6]. Previous studies mainly focused on identifying the risk factors of Toxoplasmosis but few on seropositivity, however the risk factors of seropositivity are of great importance in the study of toxoplasmosis [2,3,6,32]. Heart transplant recipients have a higher risk of transplant-transmitted toxoplasmosis, because of the high tropism of *T. gondii* in the myocardium, while toxoplasmosis is less frequent in non-heart SOT [6,30]. The prevalence of toxoplasmosis in heart transplant recipients was 1.4%, significantly higher than that in SOT patients (0.24%) [30]. For kidney transplant recipients, most cases have been reported in mismatch situations D+/R- [18,19]. A patient’s own or donor-derived toxoplasma infection both are significant potential threat to such patients. However, the serum toxoplasma antibody status of most donors was unavailable, because donor screening for *T. gondii* infection is not mandated in China so far. Although it is of significance to investigate the correlation of the serum antibody status between the donors and the recipients, the analysis was limited by lack of those data. Thus, more clinical data of donors and further studies are needed.

The impact of seropositivity on kidney recipients is of great significance, but few studies described this issue. In a study of hemodialysis patients, it was reported that patients infected with *T. gondii* had higher levels of urea and creatinine than the controls, but it did not mention in kidney transplant recipients [13]. In a recent study using Organ Procurement and Transplantation Network (OPTN) database, they found that in renal recipients *T. gondii* antibody-positive donors graft recipients have comparable survival to transplantation from *T. gondii* uninfected recipients [33]. Although the crude graft failure was similar, they did not discuss the change of renal function [33]. Our study detected no significant difference in liver and renal function tests, indicating a comparable outcome. In our study, it suggests that the infection rate of *T. gondii* in patients with uremia and post kidney transplantation were significantly higher than normal population, although the incidence of acute toxoplasmosis is significantly lower than that of subclinical infection, but this may be due to the fact that these tested patients maintained relative stable immunity, or patients with acute toxoplasma infection was not included for death and without detection. On the other hand, some immunocompromised transplant patients or uremic patients were negative for toxoplasma antibodies in our tests because of impaired humoral immunity [14,22–24].

Interestingly, we noticed that the patients with uremia, in the waiting list of kidney transplantation, had the highest seroprevalence in this study. Our analysis found it was associated with a higher percentage of raising pets, especially cats, which is a key risk factor of *T. gondii* infection [34,35]. On the contrary, kidney recipients tended to refused to raise a cat to reduce the risk of infection, although pets can provide accompany and other benefits. Thus, raising pets needs more cautions for patients with uremia to control the risk of this opportunistic infection. As is known, T cells play a critical role in the immunopathogenesis of Toxoplasma gondii infection [36,37]. We found lower percentage of CD3+T cells and CD8+T cells was associated with higher prevalence of *Tg* IgG+ in uremia patients when compared with kidney recipients, consistent with previous studies [23,38]. But we didn’t identify reduced CD4+T cells in seropositive patients. In fact, the absolute number and percentage of various immune cells are important, but the function of different immune cells, which is omitted in this study, also play a key role. Thus, more granular data would help clarify the relationship between immune status and infection risk. Meanwhile, further study is required to validate these results.

The diagnosis of *T. gondii* infection mainly depends on serological examinations, including dye test (DT), modified agglutination test (MAT), enzyme-linked immunosorbent assays (ELISA), immunosorbent agglutination assay (ISAGA), indirect fluorescent antibody test (IFAT) and indirect haemagglutination assays (IHA). Every method has its merits and demerits, but IHA is recommended for mass screening in epidemiologic surveys because of its simple and rapid operation [39]. However, IgG antibodies are produced later than IgM antibodies, so IHA IgG detection seems unable to identify acute and congenital infections, which is critical in immunocompromised populations. In addition, detection of IgG indicates the occurrence of infection without providing information about the timing of infection, which limits further evaluation of the infection.

Although this study was limited by small sample size and nature of retrospective study, but the research suggested it was important to screen *T. gondii* serology prior to transplantation, especially for those at high risk. But such a high rate of infection cannot be ignored because it is a very big risk factor. Because they may have a high infection rate of *T. gondii* pre-hospitalization, therefore there is a high risk of transforming from occult infection to acute infection after treatment.

With the advantage of broad-spectrum activity against *T. gondii*, Pneumocystis, Listeria, and Nocardia, trimethoprim/sulfamethoxazole (TMP/SMX) has become the most used chemoprophylaxis in transplant patients [6]. Studies have proved that TMP/SMX chemoprophylaxis is capable of preventing toxoplasmosis in SOT recipients, even in cases of mismatched patients (D+/R-) [6,40]. Prophylaxis is recommended for SOT patients during the post-transplant period for those at high risk [40]. However, there is no current recommendation on the use of either drug nor the duration of prophylaxis after kidney transplantation. Moreover, the prophylaxis is not capable of fully protecting against toxoplasmosis [6]. Thus, further research is necessary to find drugs and optimal the duration which are capable of preventing and curing *T. gondii* infection.

Given comparable seroprevalence and potential risk without appropriate prevention for toxoplasmosis, donor screening needs to be instituted in China. Despite limitations, we believe this study will help extend our understanding of the local epidemiology of *T. gondii* seropositivity and associated risk factors in kidney transplant recipient populations.

In conclusion, this retrospective study in central-southern China suggested that both kidney transplant recipients and uremia patients in the waiting list of kidney transplantation are more susceptible to *T. gondii* infection. Moreover, immunosuppression and the history of keeping cats were associated with seropositivity. Therefore, more caution and monitoring for surveillance of seropositivity before and after kidney transplantation are warranted, especially for those at high risk. And more study is need to validate those risk factors and to develop more effective strategy to prevent and treat *T. gondii* infection.

## References

[1] Montoya JG, Liesenfeld O. Toxoplasmosis. Lancet. 2004;363(9425):1965–76. doi: 10.1016/S0140-6736(04)16412-X 15194258

[2] Amikura T, Kikuchi T, Kato J, Koda Y, Sakurai M, Yamazaki R, et al. Toxoplasmosis after allogeneic hematopoietic stem cell transplantation: impact of serostatus-based management. Transpl Infect Dis. 2021;23(3):e13506. doi: 10.1111/tid.13506 33174304

[3] Štajner T, Vujić D, Srbljanović J, Bauman N, Zečević Ž, Simić M, et al. Risk of reactivated toxoplasmosis in haematopoietic stem cell transplant recipients: a prospective cohort study in a setting withholding prophylaxis. Clin Microbiol Infect. 2022;28(5):733.e1–733.e5. doi: 10.1016/j.cmi.2021.09.012 34555535

[4] Wesołowski R, Pawłowska M, Smoguła M, Szewczyk-Golec K. Advances and challenges in diagnostics of toxoplasmosis in HIV-infected patients. Pathogens. 2023;12(1):110. doi: 10.3390/pathogens12010110 36678458 PMC9862295

[5] Adekunle RO, Sherman A, Spicer JO, Messina JA, Steinbrink JM, Sexton ME, et al. Clinical characteristics and outcomes of toxoplasmosis among transplant recipients at two US academic medical centers. Transpl Infect Dis. 2021;23(4):e13636. doi: 10.1111/tid.13636 33993599 PMC8455410

[6] Dard C, Marty P, Brenier-Pinchart M-P, Garnaud C, Fricker-Hidalgo H, Pelloux H, et al. Management of toxoplasmosis in transplant recipients: an update. Expert Rev Anti Infect Ther. 2018;16(6):447–60. doi: 10.1080/14787210.2018.1483721 29855213

[7] Nagington J, Martin AL. Toxoplasmosis and heart transplantation. Lancet. 1983;2(8351):679. doi: 10.1016/s0140-6736(83)92553-9 6136814

[8] Robert-Gangneux F, Meroni V, Dupont D, Botterel F, Garcia JMA, Brenier-Pinchart M-P, et al. Toxoplasmosis in transplant recipients, Europe, 2010-2014. Emerg Infect Dis. 2018;24(8):1497–504. doi: 10.3201/eid2408.180045 30014843 PMC6056100

[9] Fernàndez-Sabé N, Cervera C, Fariñas MC, Bodro M, Muñoz P, Gurguí M, et al. Risk factors, clinical features, and outcomes of toxoplasmosis in solid-organ transplant recipients: a matched case-control study. Clin Infect Dis. 2012;54(3):355–61. doi: 10.1093/cid/cir806 22075795

[10] Renoult E, Biava MF, Hulin C, Frimat L, Hestin D, Kessler M. Transmission of toxoplasmosis by renal transplant: a report of four cases. Transplant Proc. 1996;28(1):181–3. 8644168

[11] Schwartz BS, Mawhorter SD, AST Infectious Diseases Community of Practice. Parasitic infections in solid organ transplantation. Am J Transplant. 2013;13 Suppl 4:280–303. doi: 10.1111/ajt.12120 23465021

[12] Zhou AL, Leng A, Ruck JM, Akbar AF, Desai NM, King EA. Kidney donation after circulatory death using thoracoabdominal normothermic regional perfusion: the largest report of the United States experience. Transplantation. 2024;108(2):516–23. doi: 10.1097/TP.0000000000004801 37691154 PMC10840803

[13] Abood MA, Saheb EJ. The effect of toxoplasmosis on renal function in hemodialysis patients. Ann Parasitol. 2022;68(4):685–92. doi: 10.17420/ap6804.475 37573499

[14] Salem DA, Hendawy SR, Nassar MK. Seroprevalence of *Toxoplasma gondii* among hemodialysis patients: a possible link to main T-lymphocyte subsets levels and dialysis adequacy. Acta Trop. 2023;237:106703. doi: 10.1016/j.actatropica.2022.106703 36181878

[15] Foroutan M, Rostami A, Majidiani H, Riahi SM, Khazaei S, Badri M, et al. A systematic review and meta-analysis of the prevalence of toxoplasmosis in hemodialysis patients in Iran. Epidemiol Health. 2018;40:e2018016. doi: 10.4178/epih.e2018016 29748456 PMC6060338

[16] Soltani S, Kahvaz MS, Soltani S, Maghsoudi F, Foroutan M. Seroprevalence and associated risk factors of *Toxoplasma gondii* infection in patients undergoing hemodialysis and healthy group. BMC Res Notes. 2020;13(1):551. doi: 10.1186/s13104-020-05396-5 33287882 PMC7720589

[17] Arab-Mazar Z, Fallahi S, Yadegarynia D, Javadi Mamaghani A, Seyyed Tabaei SJ, Rajaeian S, et al. Immunodiagnosis and molecular validation of *Toxoplasma gondii* infection among patients with end-stage renal disease undergoing haemodialysis. Parasitology. 2019;146(13):1683–9. doi: 10.1017/S0031182019001033 31397237

[18] Gay J, Gendron N, Verney C, Joste V, Dardé M-L, Loheac C, et al. Disseminated toxoplasmosis associated with hemophagocytic syndrome after kidney transplantation: a case report and review. Transpl Infect Dis. 2019;21(5):e13154. doi: 10.1111/tid.13154 31373746

[19] Francí EV, Adekunle RO, Nucci M, Pouch SM. Complex considerations - fever and pancytopenia after solid organ transplantation. Transpl Infect Dis. 2023;25(4):e14079. doi: 10.1111/tid.14079 37279241

[20] Kotton CN. Zoonoses in solid-organ and hematopoietic stem cell transplant recipients. Clin Infect Dis. 2007;44(6):857–66. doi: 10.1086/511859 17304461

[21] Rasti S, Hassanzadeh M, Soliemani A, Hooshyar H, Mousavi SGA, Nikoueinejad H, et al. Serological and molecular survey of toxoplasmosis in renal transplant recipients and hemodialysis patients in Kashan and Qom regions, central Iran. Ren Fail. 2016;38(6):970–3. doi: 10.3109/0886022X.2016.1172940 27097530

[22] Soltani Tehrani B, Mirzajani E, Fallahi S, Manouchehri Naeini K, Mahmoudi MR, Safari Kavishahi M, et al. Challenging TaqMan probe-based real-time PCR and loop-mediated isothermal amplification (LAMP): the two sensitive molecular techniques for the detection of toxoplasmosis, a potentially dangerous opportunistic infection in immunocompromised patients. Arch Microbiol. 2020;202(7):1881–8. doi: 10.1007/s00203-020-01903-1 32448961

[23] Betjes MGH. Immune cell dysfunction and inflammation in end-stage renal disease. Nat Rev Nephrol. 2013;9(5):255–65. doi: 10.1038/nrneph.2013.44 23507826

[24] Liu Q, Wang Z-D, Huang S-Y, Zhu X-Q. Diagnosis of toxoplasmosis and typing of *Toxoplasma gondii*. Parasit Vectors. 2015;8:292. doi: 10.1186/s13071-015-0902-6 26017718 PMC4451882

[25] Adekunle RO, Sherman A, Spicer JO, Messina JA, Steinbrink JM, Sexton ME, et al. Clinical characteristics and outcomes of toxoplasmosis among transplant recipients at two US academic medical centers. Transpl Infect Dis. 2021;23(4):e13636. doi: 10.1111/tid.13636 33993599 PMC8455410

[26] Dard C, Marty P, Brenier-Pinchart M-P, Garnaud C, Fricker-Hidalgo H, Pelloux H, et al. Management of toxoplasmosis in transplant recipients: an update. Expert Rev Anti Infect Ther. 2018;16(6):447–60. doi: 10.1080/14787210.2018.1483721 29855213

[27] Li Z, Yi H, Zheng X, Zhu Y, Lu B, Zhang N, et al. *Toxoplasma gondii* infection is associated with schizophrenia from the perspectives of seroepidemiology and serum metabolomics in Hunan Province, China. Microb Pathog. 2024;195:106880. doi: 10.1016/j.micpath.2024.106880 39181191

[28] Gan D, Fan J, Zeng J, Lin J, Chen X. Seroprevalence of the specific antibody against *Toxoplasma gondii* among patients with hematological diseases. Zhongguo Xue Xi Chong Bing Fang Zhi Za Zhi: Chinese J Schistosomiasis Control. 2023;36(1):83–6. doi: 10.16250/j.32.1374.2023197 38604690

[29] Li Z, Lei Z, Yang W, Jing C, Sun X, Yang G, et al. Anti-*Toxoplasma gondii* antibodies as a risk factor for the prevalence and severity of systemic lupus erythematosus. Parasit Vectors. 2024;17(1):44. doi: 10.1186/s13071-024-06141-8 38291478 PMC10826107

[30] Robert-Gangneux F, Sterkers Y, Yera H, Accoceberry I, Menotti J, Cassaing S, et al. Molecular diagnosis of toxoplasmosis in immunocompromised patients: a 3-year multicenter retrospective study. J Clin Microbiol. 2015;53(5):1677–84. doi: 10.1128/JCM.03282-14 25762774 PMC4400795

[31] Kanno Y, Okamoto K, Shinohara T, Kinoshita O, Hatano M, Ikeda M, et al. Pre-transplant seroprevalence, associated factors, and post-transplant incidence of *Toxoplasma gondii* infection among heart transplant recipients in Japan. Transplant Proc. 2024;56(1):148–52. doi: 10.1016/j.transproceed.2023.11.015 38177043

[32] Galván-Ramírez ML, Sánchez-Orozco LV, Andrade-Sierra J, Mendoza-Cabrera S, Evangelista-Carrillo LA, Rodríguez Pérez LR, et al. Toxoplasma infection in kidney donors and transplant recipients from Western Mexico: a one-year follow-up. Transpl Infect Dis. 2019;21(5):e13139. doi: 10.1111/tid.13139 31271696

[33] Butani L, Tancredi D. Outcomes of kidney transplants from Toxoplasma-positive donors: an organ procurement and transplant network database analysis. Transpl Int. 2024;37:13203. doi: 10.3389/ti.2024.13203 39055345 PMC11269094

[34] Hofer U. The cat is out the bag about Toxoplasma host range. Nat Rev Microbiol. 2019;17(11):646–7. doi: 10.1038/s41579-019-0266-6 31485033

[35] Matta SK, Rinkenberger N, Dunay IR, Sibley LD. *Toxoplasma gondii* infection and its implications within the central nervous system. Nat Rev Microbiol. 2021;19(7):467–80. doi: 10.1038/s41579-021-00518-7 33627834

[36] Gazzinelli RT, Hakim FT, Hieny S, Shearer GM, Sher A. Synergistic role of CD4+ and CD8+ T lymphocytes in IFN-gamma production and protective immunity induced by an attenuated *Toxoplasma gondii* vaccine. J Immunol. 1991;146(1):286–92. doi: 10.4049/jimmunol.146.1.286 1670604

[37] Suzuki Y, Remington JS. Dual regulation of resistance against *Toxoplasma gondii* infection by Lyt-2+ and Lyt-1+, L3T4+ T cells in mice. J Immunol. 1988;140(11):3943–6. doi: 10.4049/jimmunol.140.11.3943 3259601

[38] Shehata AI, Hassanein F, Abdul-Ghani R. Opportunistic parasitoses among Egyptian hemodialysis patients in relation to CD4+ T-cell counts: a comparative study. BMC Infect Dis. 2019;19(1):480. doi: 10.1186/s12879-019-4110-4 31142275 PMC6542030

[39] Caruana LB. A study of variation in the indirect hemagglutination antibody test for toxoplasmosis. Am J Med Technol. 1980;46(6):386–91. 7386515

[40] Gourishankar S, Doucette K, Fenton J, Purych D, Kowalewska-Grochowska K, Preiksaitis J. The use of donor and recipient screening for toxoplasma in the era of universal trimethoprim sulfamethoxazole prophylaxis. Transplantation. 2008;85(7):980–5. doi: 10.1097/TP.0b013e318169bebd 18408578

